# Predictors of Risk of Muscle Injury in Non-Professional Soccer Players: An Ambispective Cohort Study

**DOI:** 10.3390/sports12110314

**Published:** 2024-11-20

**Authors:** Ismael Iraqui-Rato, Rubén Cuesta-Barriuso

**Affiliations:** 1 Department of Surgery and Medical-Surgical Specialties, University of Oviedo, 33003 Oviedo, Spain; ismaeliraqui@gmail.com; 2 InHeFis Research Group, Instituto Asturiano de Investigación Sanitaria (ISPA), 33011 Oviedo, Spain

**Keywords:** soccer, muscle injury, risk, secondary prevention, physiotherapy

## Abstract

(1) Background: Soccer accounts for 30% of all sports injuries. Muscle injuries in soccer, not caused by trauma or contact, are the most common. The objective was to assess the risk of injury based on the footwear used by soccer players and the playing field and to identify the best predictive model of muscle injuries; (2) Methods: An ambispective cohort study. The primary variable was the number of muscle injuries in the last three seasons. The secondary variables were age, body mass index, type of soccer shoes and turf, training load and position on the field. The possible confounding variables were motivation for the choice of footwear, date of injuries, time playing and regular first-team player status; (3) Results: 156 players were recruited. The risk of injury is 1.03 (95% CI: 0.83; 1.27) times more frequent in players competing on artificial turf. The risk of injury is slightly higher in first-team players than in substitutes (RR = 1.02; 95% CI: 0.79; 1.32). There was no statistically significant relationship between the position on the field (*p* = 0.91), the type of shoe (*p* = 0.69) and the motivation to buy the shoes (*p* = 0.82), regarding the risk of muscle injury in these athletes. The best model to estimate risk of injury includes age, training load and soccer shoe type as confounding variables (AIC = 190.5; *p* = 0.10; χ^2^_(3)_ = 10.14; *p* = 0.02); (4) Conclusions: The risk of muscle injury is higher in non-professional soccer players competing on artificial pitches and in those who are regular starters on their teams. Field position, soccer shoe and motivation to purchase the soccer shoe are variables that do not increase the risk of muscle injury in these athletes. The best predictive model of injury includes age, training load and shoe type.

## 1. Introduction

Both professional and amateur soccer subject players to physical demands that increase the risk of musculoskeletal injuries [1]. The injury rates are varied in the literature, showing a fluctuation, during matches, from 12 to 66 injuries per 1000 h of play. During the workouts, the described rates vary from 1.5 to 7.6 injuries per 1000 h [2,3]. The incidence of injuries during competition is significantly higher, being approximately 4–6 times higher than during training [4].

Ankle, knee, hamstring, and groin injuries are the most common, accounting for more than 50% of all injuries in soccer, although more than 65% are classified as minor [5,6].

The biomechanics of soccer and its interaction with the pitch are crucial aspects that can influence the incidence of injuries in these athletes. Muscle and tendon injuries are more prevalent in amateur soccer players than in professionals (4.78%). Likewise, moderate, and serious injuries are more frequent in amateur players than in professional athletes, by 9.60% [5,6]. The maintenance of soccer pitches, which varies between professional and amateur sport mainly for reasons of financial resources, must be taken into account. This may suggest that different playing surfaces may have a significant impact on the biomechanical load experienced by players. In this regard, artificial turf, despite its technological improvements, is still associated with an increased risk of musculoskeletal injuries, such as sprains and strains [7]. A recent study [8] has described the relationship between players’ positions on the field and knee pain, with forwards experiencing more pain, and found no association between players’ body mass index and the occurrence of sports injuries. Variables such as age, time spent in strenuous activity, timing of puberty and overweight/obesity have been associated with an increased risk of anterior cruciate ligament injury in young adults and adolescents [9]. However, in non-professional soccer players, the variables that affect the risk of muscle injury have not yet been described beyond fatigue, overload, and intensity variables.

A proper choice of footwear is essential to prevent injuries in soccer players and improve their performance on the pitch [10]. The type of footwear, including the design of the cleats and the sole, can influence the biomechanics of the players and their susceptibility to the development of injuries [11]. In this regard, shoes with round cleats have been shown to be safer than blade-type cleats with respect to the development of torsion injuries [12]. However, in non-professional soccer players, the choice of footwear is not determined by sponsors, as the cost is borne by the player himself, and often the choice of footwear is based on price, comfort or the model of boot. It has been described how the incidence of injuries on natural grass is 57.78%, higher than the 42.22% registered on artificial turf [13]. However, despite the relevance of this choice, players may not adequately consider these factors when selecting their footwear, which may increase the risk of muscle injuries.

The aim of this study was to evaluate the risk of muscle injuries in non-professional soccer players and identify the best predictive model of muscle injuries based on anthropometric and sports variables.

## 2. Materials and Methods

### 2.1. Study Design

An ambispective cohort study.

### 2.2. Participants

The inclusion criteria were soccer players that (i) were non-professional, who competed in a regional territorial area (Principality of Asturias, Spain); (ii) had not undergone musculoskeletal surgery in the three seasons prior to the study; and (iii) had federated at least one year before the study period. The exclusion criteria were (i) those who had not competed, during the study seasons, for a period of more than 6 months due to a musculoskeletal injury.

### 2.3. Ethical Considerations

This study took into account the ethical aspects contained in the Declaration of Helsinki. The research project was approved by the Research Ethics Committee of the Principality of Asturias (CEImPA code 2024.107). The study was registered in the international database of clinical records, www.clinicaltrials.gov (NCT06425809).

### 2.4. Study Variables

Data collection was carried out in May 2024. This was conducted through a closed-question format interview. Data collection was carried out at the regional soccer federation using a self-reported closed-response survey model, with all interviews taking place in the same week. The primary variable was the number of lower limb muscle injuries in the last three seasons.

The secondary and modifying variables were age (years), body mass index (kg/m^2^), type of training and competition shoe (rubber/metal cleat), training and competition playing field (sand/artificial turf/natural turf), type of muscle injury (fibrillar rupture/tendon muscle/strain), weekly training load (hours) and position on the field (goalkeeper/defender/midfielder/forward). The possible confounding variables were the motivation for the choice of footwear (comfort/aesthetics/price/other), the date of muscle injuries, the time competing in the category (in completed months) and the usual presence on the starting team (yes/no). These confounding variables were selected because of their influence on a possible misinterpretation of the results due to a false association between injury risk and possible modifying variables

### 2.5. Sample Size

The calculation of the sample size was carried out using the statistical program Stata IC16 (StataCorp LLC, College Station, TX, USA). This analysis estimated a prevalence of muscle injuries of 0.05 (5%). By calculating for amplitudes from 0.05 to 0.80 (delta = 0.02), a percentage of 5% of injury cases was expected. Using the proportion 0.05 (SD = 0.50), the sample size was calculated for effects between 0.01 and 0.8 (delta = 0.02). With these criteria, the sample size to estimate this prevalence with an accuracy of ±2% was 97 non-professional soccer players from the Principality of Asturias. The estimation of the external validity of the predictive model required a 40% larger sample. Thus, a sample size of at least 137 soccer players was estimated.

### 2.6. Statistical Analysis

The statistical analysis was performed with the statistical program Stata IC16 (StataCorp LLC). The statistical significance was estimated at α = 0.05. Using a double-entry table (2 × 2), the prevalence of adverse effects in soccer players with muscle injuries in the study period was calculated. The relative risk (RR), the risk difference (RD) and the risk difference in the population (RDp) of muscle injuries in these athletes were also calculated.

The influence of possible confounding factors was also calculated by discarding those whose RR was in the range 0.67–1.50. Any variable that, when adjusted, caused changes in RR > 10% was considered a confounding factor.

Through a logistic regression analysis, the best predictive model was selected based on the AIC index and the reclassification indices, selecting the most parsimonious model, calculating the Mantel–Haenszel weighting (RRMH). The multicollinearity of the predictor variables was calculated using the variance inflation factor (VIF). The Wald test was used to check if the estimated RRs in the strata of the possible confounding factors were homogeneous.

The predictive capacity was calculated with the validity indices (area under the curve—AUC, sensitivity and specificity). With the probability of suffering a muscle injury, calculated with the logistic regression model for the athletes included in the study, the risk of injury for four athletes was calculated from the OR of the estimated model.

## 3. Results

### 3.1. Descriptive Analysis

The study included 156 soccer players. The mean age was 24.03 (SD: 4.29) years, referring to an average of 1.46 (SD: 1.66) muscle injuries throughout the three evaluation seasons. Most of the players had suffered a previous muscle injury (68.59%). Artificial turf soccer fields were the most common in training (90.38%) and in competition (52.56%). Rubber cleats were the most used (76.92%) by the players. Table 1 shows all the results of the descriptive analysis of the sociodemographic, anthropometric and sports variables of the players included in the study.

### 3.2. Analysis of the Risk of Muscle Injury

The risk of injury is 1.03 times more frequent in players who compete on an artificial field than in those who train on natural turf fields (RR = 1.03 [0.83; 1.27]). Players who compete on artificial fields exhibit 2% more injuries than those who usually play on natural grass (RD = 0.02). For every 1000 injuries in federated soccer players, 10 injuries occur due to competing on artificial turf fields (RDp = 0.01). Three percent of all injuries of soccer players playing on artificial turf are due to this circumstance (AFe = 0.03). One percent of the total muscle injuries that occur in federated soccer players are caused by competing on artificial fields on a regular basis (AFp = 0.01).

The risk of injury is 1.02 times higher in starting players than in substitutes (RR = 1.02 [0.79; 1.32]). There are 1.4% more injuries in first-team players than in those who do not usually play (RD = 0.014). For every 1000 injuries in federated soccer players, there are four injuries due to playing as first-team players (RDp = 0.004). Two percent of all injuries of regular starting soccer players are due to this circumstance (AFe = 0.02). Only 0.6% of the total muscle injuries in federated soccer players are due to the presence on the starting team on a regular basis (AFp = 0.006).

Lastly, there was no statistically significant relationship between the position on the field (*p* = 0.91), the type of shoe (*p* = 0.69) and the motivation to buy the shoes (*p* = 0.82), regarding the risk of muscle injury in these athletes. Table 2 shows the results of the risk analysis depending on the training and competition field, regular presence as first-team player and the nominal qualitative variables.

### 3.3. Analysis of the Association Between the Type of Turf and First-Team Player Status

The association analysis shows that first-team player status is not a modifier of the effect of the type of soccer pitch (natural or artificial) on the development of muscle injuries. There are no statistically significant differences for the type of training turf (χ^2^_(1)_ = 2.75; *p* = 0.09) and competition turf (χ^2^_(1)_ = 0.50; *p* = 0.48), depending on whether or not the athlete is a regular first-team player. Thus, first-team player status in the soccer team is not a modifier of the effect. Table 3 shows the analysis of the association between the development of injuries and first-team player status, stratified by the regular presence on the team.

### 3.4. Building the Best Predictive Model

The selected model (AIC = 190.5) contains three predictors of muscle injury in soccer players: age, training load and shoe type. The predictive capacity of this model was compared with the following model that included one more predictor by means of reclassification indices (IDI = 0.02%; *p* = 0.10; NRI = 0.04%; *p* = 0.11). The analysis showed no differences in the results, choosing the selected model, being more parsimonious, as the best predictive model of risk of injury.

The capacity of the predictive model was estimated (χ^2^_(3)_ = 10.14; *p* = 0.02). When performing the multicollinearity analysis of the predictor variables, we observed how the variables are moderately correlated with each other (mean VIF = 1.12). The odds ratios obtained in the predictive model reflect that the risk of injury increases with age (OR = 1.11). As for the type of soccer shoe used, the use of rubber cleats presents the greatest risk, as well as aluminum cleats (OR = 1), while the use of multi-cleat boots presents a protective effect against suffering muscle injury (OR = 0.44; 95% CI = 0.08; 1.10). The training load does not increase the risk of injury (OR = 0.85; 95% CI = 0.72; 1.00). The results of the analysis of the predictive model with a linear regression model are shown in Table 4.

### 3.5. Validity Indices of the Selected Model

**Figure 1:**
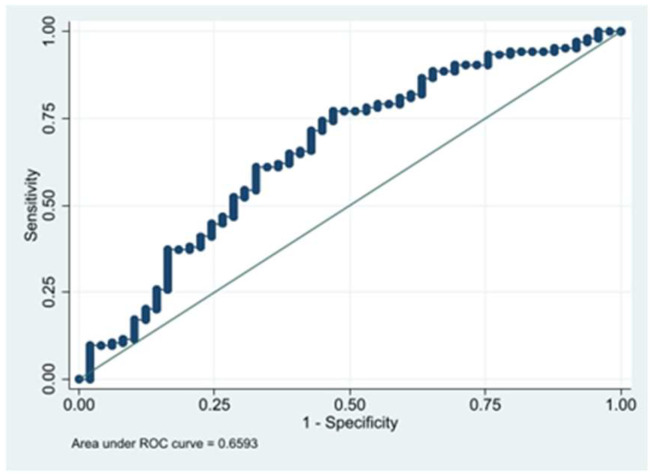
Area under the curve calculated in the predictive model.

After calculating the validity indices of the model, a moderate overall predictive capacity was revealed (AUC = 0.66). For the cut-off point *p* = 0.5, it has a high predictive capacity to detect athletes with muscle injury (sensitivity [Se] of 94.3%), but a low capacity to detect subjects without injury (specificity [Sp] = 14.3%). To obtain a given sensitivity and specificity of the model, the most indicated cut-off point was estimated as closely as possible (*p* = 0.66; Se = 73.33%; Sp = 55.10%). Figure 1 shows the area under the curve calculated in the selected predictive model.

### 3.6. Assessment of Interaction and Confusion

Evaluation of the interaction was carried out to quantify the effect of the type of training turf on the risk of injury, with the three predictors of the model (age, training load and type of soccer shoe), using the likelihood ratio test. There was statistical significance of the set of interactions (−2ln LR = 11.83; *p* = 0.003). The result indicates that the interactions between the type of training turf and the type of soccer shoe and the training load are statistically significant (*p* < 0.01). When assessing confusion, there are changes of more than 10% in all the reduced models. When analyzing the interaction to quantify the effect of the type of competition turf on the risk of injury, with the three predictors of the model (age, training load and type of soccer shoe), there was no statistical significance of the set of interactions (−2ln LR = 2.17; *p* = 0.54). Thus, the best model to estimate the effect of the competition turf on the development of injuries includes age, training load and shoe type as confounding variables.

## 4. Discussion

The present study aimed to evaluate the risk of muscle injuries in non-professional soccer players and identify the best predictive model of muscle injuries. Likewise, the risk of developing muscle injuries in soccer players was analyzed depending on the footwear, the playing field, age, body mass index, weekly training load and position on the field.

Although the risk of muscle injury is more frequent in players who train on artificial turf and those who are usually starters in their respective teams, there are hardly any statistically significant differences. The best predictive model of muscle injuries in these athletes determined how age, weekly training load and shoe type are the most significant predictive variables of risk of injury. These findings underline the complexity of the factors that contribute to injuries in soccer, suggesting that both environmental conditions and the individual characteristics of players should be considered when designing injury prevention programs.

The analysis of the risk of injury depending on the type of turf showed how players competing on artificial turf have 13% fewer injuries compared to those playing on natural grass. This finding could be explained by the uniformity and consistency of artificial turf, thereby being able to reduce unexpected variations of the surface. This could justify the decrease in injuries resulting from sudden changes in the terrain. Artificial surfaces provide more constant traction and cushioning, which can reduce joint and muscle stress during the execution of rapid movements and changes of direction. These results coincide with previous studies where a lower incidence of injuries has been reported in sports practice on artificial turf due to its uniform design and greater durability [14,15]. However, the results of other studies should be considered [16,17,18] where an association has been observed between playing on artificial turf and certain types of injuries, such as skin abrasions and specific muscle problems.

Our results found no association between the risk of muscle injuries and regular first-team player status. The training load and exposure to risk during matches is similar in starters and substitutes, regardless of how frequently they play [19]. Our results are consistent with what was observed in previous studies [15,20] reporting that first-team player status is not a determining factor in the incidence of injuries in soccer players. In addition, other factors such as adequate post-match recovery, fatigue management and player rotation strategies can play a relevant role that helps mitigate the risk of injuries [21,22,23]. Team management and training tactics must be optimized to protect players equally, regardless of the first-team player status of athletes.

The absence of an association between injuries and the motivation for buying the footwear, the position on the field or the type of soccer shoe revealed how these variables do not significantly influence the risk of injury. This may suggest how intrinsic factors such as physical preparation, general health status and playing technique have a greater impact on injury prevention than the specific equipment used. These results have been described previously [15,24,25], indicating how individual characteristics and general preparation are more crucial for injury prevention than other extrinsic variables, such as the type of soccer shoe used or the position on the pitch. In addition, personal preferences and comfort with equipment can vary considerably between players, making it difficult to establish a direct relationship between the type of soccer shoe and the risk of injury. It is possible that other factors, such as individual biomechanics and adaptation to the type of shoe, play a more important role in the incidence of injuries [26,27,28]. In this way, coaches, trainers and health personnel of the teams should focus on evaluating and optimizing the physical and technical condition of each player, instead of focusing excessively on the type of equipment.

We have observed in our predictive model how the training load is a predictive factor of risk of injury. The more players train, the lower is their risk of injury. This relationship may indicate how better physical conditioning and progressive adaptation of the body to exertion prevents the development of injuries [29]. Therefore, a well-planned training load can allow a gradual progression in the intensity and volume of the sessions, increasing the effectiveness in muscle strengthening and endurance, thus reducing the susceptibility to injuries [30].

The predictive model also showed that younger players have a lower risk of injury. This protective effect may be due to the greater recovery capacity and the lower accumulated wear of younger athletes [31]. Younger age is associated with greater muscle plasticity and a faster ability to adapt to the physical demands of sport [32]. However, looking at the age range of the athletes included in the study, this variability could be because older players do less exercise due to their status on the team, where they may be more established due to their experience.

Finally, the use of multi-cleat boots was associated with a lower risk of injury. This can probably be due to offering a better pressure distribution and greater stability [33]. These types of boots can reduce the risk of sprains and other joint problems by providing a more solid and secure grounding during rapid movements and changes of direction. The inclusion of the type of soccer shoe in the predictive model of the risk of developing muscle injury is in agreement with previous studies where the importance of using specially designed equipment to reduce the risk of injuries is underlined [34,35].

The findings of this study may be relevant to sports professionals. The identification of the most relevant risk factors as well as the predictive model of injury in this population of football players may help in the implementation of more effective and personalized prevention strategies. In this way, it could contribute to reduce the incidence of injuries and improve the performance of these players. These results are also in line with the importance of conducting regular assessments and adjusting prevention strategies according to the individual needs of the players, which could include changes in equipment, modification of training regimes and incorporation of advanced recovery techniques.

### Limitations of the Study

The collection of data through self-reported surveys can introduce a bias in the collection of information. The tendency of players to minimize or exaggerate their injury and training experiences can modify the analyzed data. The absence of a thorough control in the evaluation of variables such as intensity and the specific type of training can affect the results and the accuracy of the conclusions. Similarly, a relevant limitation of this study is the non-inclusion of variables such as the weekly competition load (in hours), muscle overload and fatigue, or the intensity of the training or competition (distance covered, heart rate, etc.). This limits the interpretation and generalization of the results of this study. It is possible that different training programs, with variations in intensity, duration, and type of exercise, may significantly affect the results, something that could not be controlled in this study. Therefore, future studies should collect data through direct monitoring of training. Similarly, as only male soccer players were included, these results cannot be generalized to female amateur soccer players. Although female professional soccer players show injury incidence rates and patterns comparable to those of male players [36], in non-professional female soccer players, these data need to be confirmed. Specific studies on female soccer players or comparing both sexes could help to identify risk factors more globally and whether sex influences the risk of muscle injury in these non-professional athletes.

## 5. Conclusions

The risk of injury is higher for non-professional players competing on artificial turf. This risk of muscle injury is also higher in players who are regular starters in their football teams than in substitutes. Field position, boot type and motivation to buy the boot are not related to an increased risk of muscle injury in these non-professional soccer players.

The best predictive model of risk of injury in non-professional soccer players includes age, training load and shoe type.

## Figures and Tables

**Table 1:** Descriptive analysis (mean, standard deviation and 95% confidence interval) of the characteristics of the soccer players included in the study.

Variables		Mean (Standard Deviation)	[95% CI]
	Age (years)	24.03 (4.29)	[23.35; 23.47]
Anthropometrics	Weight (kg)	73.73 (8.05)	[72.46; 75.01]
	Height (cm)	177.96 (7.06)	[176.84; 179.08]
	Body mass index (kg/m^2^)	23.22 (1.56)	[22.98; 23.47]
Injuries	Total injuries (number)	1.46 (1.66)	[1.20; 1.72]
	Quadriceps (number)	0.33 (0.61)	[0.23; 0.42]
	Hamstrings (number)	0.70 (1.04)	[0.53; 0.86]
	Gastrocnemius (number)	0.16 (0.43)	[0.09; 0.23]
Sports	Weekly training (hours)	6.75 (2.17)	[6.41; 7.10]
	Individual training (hours)	3.10 (2.76)	[2.64; 3.52]
	Time competing in the category (months)	14.39 (4.56)	[13.67; 15.11]
		n (%)	
Previous muscle injuries	Yes	107 (68.59)	
	No	49 (31.41)	
Type of muscle injury	Fibrillar rupture	99 (63.46)	
	Tendon muscle	51 (32.69)	
	Strain	6 (3.85)	
Usual presence on the starting team	Yes	123 (78.85)	
	No	33 (21.15)	
Position on the field	Goalkeeper	12 (7.69)	
	Defender	63 (40.38)	
	Midfielder	53 (33.97)	
	Forward	28 (17.95)	
Training playing field	Natural turf	15 (9.62)	
	Artificial turf	141 (90.38)	
Competition playing field	Natural turf	74 (47.44)	
	Artificial turf	82 (52.56)	
Type of training and competition shoe	Rubber cleat	120 (76.92)	
	Multi-cleat	34 (21.79)	
	Metal cleat	2 (1.28)	
Motivation for choosing a football boot	Comfort	87 (55.77)	
	Price	24 (15.38)	
	Playing field	45 (28.85)	

**Table 2:** Injury risk analysis in the soccer players included in the study.

Variables		Injury		R [95% CI]	RR	RD	RDp	AFe	AFp
		Yes	No						
Usual presence on the starting team	Starting players	84	39	0.70 [0.54; 0.85]	1.02 [0.79; 1.32]	−0.014	−0.004	0.02	0.006
	Substitutes	23	10	0.68 [0.60; 0.76]					
Training playing field	Natural turf	12	3	0.80 [0.60;1.00]	0.84 [0.64; 1.11]	0.13	0.04	0.16	0.05
	Artificial turf	95	46	0.67 [0.59; 0.75]					
Competition playing field	Natural turf	50	24	0.68 [0.56; 0.77]	1.03 [0.83; 1.27]	−0.02	−0.006	0.03	0.01
	Artificial turf	57	25	0.70 [0.58; 0.78]					
Position on the field	Goalkeeper	8	4	0.67 [0.39; 0.86]	z = 0.12; *p* = 0.91				
	Defender	43	20	0.68 [0.56; 0.78]					
	Midfielder	35	18	0.66 [0.53; 0.77]					
	Forward	21	7	0.75 [0.57; 0.87]					
Type of training and competition shoe	Rubber cleat	84	36	0.70 [0.61; 0.77]	z = −0.40; *p* = 0.69				
	Multi-cleat	21	13	0.61 [0.45; 0.76]					
	Metal cleat	2	0	1.00 [0.34; 1.00]					
Motivation for choosing a football boot	Comfort	59	28	0.68 [0.57; 0.77]	z = −0.23; *p* = 0.82				
	Price	19	5	0.79 [0.60; 0.91]					
	Playing field	29	16	0.64 [0.50; 0.77]					

R: risk; 95% CI: 95% confidence interval; RR: relative risk; RD: risk difference; RDp: risk difference in the population; AFe: attributable fraction to the exposed; AFp: attributable fraction in the population.

**Table 3:** Association between the development of muscle injuries and the playing field in training and competition, stratified by presence on the regular team.

Action	Usual Presence on the Starting Team	R [95% CI]	RR	IS	ES	RR_wV_	RR_M-H_
Training	Yes	0.85 [0.63; 1.15]	0.84	0.81	0.84	0.74	0.84
	No	0.69 [0.54; 0.87]					
Competition	Yes	1.07 [0.84; 1.36]	1.03	1.02	1.03	1.02	1.03
	No	0.89 [0.57; 1.39]					

R: risk; 95% CI: 95% confidence interval; RR: relative risk; IS: internal standardization; ES: external standardization; RR_wV_: weighting by the inverse of the variance; RR_M-H_: weighting of Mantel–Haenszel.

**Table 4:** Analysis of the predictive model with a linear regression model.

Injury	OR	SE	z	*p*	95% CI
Age	1.11	0.06	2.06	0.04	1.01; 1.23
Weekly training	0.85	0.07	−1.91	0.06	0.72; 1.00
Soccer shoe					
Multi-cleat	0.44	0.20	−1.77	0.08	0.18; 1.10
Metal cleat	1				
_const	0.65	0.87	−0.32	0.75	0.05; 8.81

OR: odds ratio; SE: standard error; 95% CI: 95% confidence interval.

## Data Availability

The data that support the findings of this study are available on request from the corresponding author. The data are not publicly available due to privacy or ethical restrictions.
